# The pelvis urinary microbiome in patients with kidney stones and clinical associations

**DOI:** 10.1186/s12866-020-01992-4

**Published:** 2020-11-05

**Authors:** Fengping Liu, Nan Zhang, Yunhong Wu, Peng Jiang, Tingting Jiang, Yang Wang, Yuwei Zhang, Qixiao Zhai, Yeqing Zou, Ninghan Feng

**Affiliations:** 1 Wuxi School of Medicine, Jiangnan University, Wuxi, 214122 China; 2 Department of Urology, Affiliated Wuxi No.2 Hospital, Nanjing Medical University, Wuxi, 214002 China; 3 LC-Bio Technology Co, Ltd, Hangzhou, 310000 China; 4 State Key Laboratory of Food Science and Technology and School of Food Science and Technology, Jiangnan University, Wuxi, 214122 China; 5 Basic Medical School, Jiangsu Vocational College of Medicine, Yancheng, 224000 China

**Keywords:** Bladder urinary microbiome, Blood microbiome, Kidney pelvis urinary microbiome, Kidney stone, Kidney function

## Abstract

**Background:**

The long-held notion that, without urinary tract or circulatory infection, bladder urine and blood are sterile biofluids has been disproven. There have been no previous reports on the kidney pelvis urinary microbiome after bladder disinfection in kidney stone patients. This study aimed to determine whether a kidney pelvis urinary microbiome is present after eliminating the influence of the bladder urinary microbiome, whether the microbiome composition is different in patients with stone kidney pelvis (SKP) and non-stone kidney pelvis (NSKP), and the correlation between SKP and patient clinical characteristics.

**Results:**

Comparisons of bacterial diversity and community structure exhibited that urine in bladder was similar to SKP and NSKP. However, the comparisons showed that urine samples were different from blood. The most common operational taxonomic units were shared by all three types of urine samples. *Corynebacterium* was significantly higher in SKP compared to NSKP. Several bacteria were associated with patient characteristics, including *Lactobacillus*, which was positively correlated with fasting blood glucose, and *Prevotella* was negatively correlated with BMI. *Lactobacillus* was significantly higher in SKP compared to blood but not in NSKP compared to blood.

**Conclusions:**

The composition of the kidney pelvis urinary microbiome after disinfection of the bladder and its similarity to the bladder microbiome indicate that bladder urine can be used to replace kidney pelvis urine in microbiome research. Additionally, the comparison of SKP and NSKP and clinical associations suggest that the occurrence of kidney stones is responsible for the SKP urinary microbiome.

## Background

Kidney stones affect approximately one in 11 individuals in their lifetimes, and their prevalence is increasing [1]. The recurrence rate within the first 5 years of the initial episode is as high as 50% [2], and some patients may develop chronic kidney disease and end-stage renal disease [3]. Despite its high incidence and severe complications, the pathophysiologic mechanisms of kidney stone formation remain incompletely characterized. Previous research has shown that kidney stones are mainly attributed to dietary patterns [4]. However, in recent decades, emerging evidence has suggested that gut microbiome dysbiosis is linked to the pathophysiology of kidney stones [5–7]. The microbiome composition in patients with kidney stones exhibited lower biodiversity and underrepresentation of some taxa compared with that observed in healthy controls [5]. In addition, alterations were linked to changes in functionality contributing to kidney stone physiopathology [5].

Although the bladder was previously considered to be “sterile”, it has been shown to possess its own urinary microbiome [8, 9]. The gut microbiome affects human gut health; similarly, the bladder urinary microbiome is also related to urinary tract diseases [10–12]. Recently, Dornbier and colleagues compared the upper and lower tract urine of kidney stone patients and obtained evidence of an upper tract microbiome; the authors found that there was no significant difference in the bacterial community between the upper and lower tract urine [13]. However, it is still unclear whether there is a bacterial community in kidney pelvis urine after bladder disinfection. Similar to the situation regarding the bladder, the dogma regarding the sterility of the human circulatory system has been disproven [14–16], and the blood microbial profile has been associated with kidney function [16]. The occurrence of kidney stones is associated with mutations in genes encoding epithelial cell tight junctions (e.g., claudin-14, an integral membrane protein that provides barrier function), which permit selective paracellular transport from the circulatory system to the kidneys [17]. This may contribute to the alteration of urinary components, such as elevated urine calcium and oxalate [18, 19], and decreased urine citrate excretion [20], which may affect the environment of microorganisms in the kidney pelvis. Given the close anatomical relationship between the kidney pelvis and bladder and the differences in kidney function and tight junctions in patients with kidney stones, we hypothesize that the existence of a urinary microbiome in the kidney pelvis is associated with the bladder urinary microbiome and blood microbiome. In addition, we hypothesized that the urinary microbiome in the kidney pelvis is different between kidneys with and without stones because of the presence of microbiomes in urinary stones as reported by Dornbier et al. [13]. Furthermore, we hypothesized that the clinical characteristics of patients are affected by the composition of the urinary microbiome in the kidney pelvis.

## Results

### Clinical parameters and expanded quantitative urine culture (EQUC)

Fifty patients with unilateral kidney stones who were negative on EQUC in post-iodophor bladder lavage urine were recruited for the study. Table 1 shows that the percentages of patients with elevated blood uric acid (BUA) and blood urea nitrogen (BUN) were 24 and 20%, respectively. The percentage of patients with reduced estimated glomerular filtration rate (eGFR) was 48%. Furthermore, Table 1 illustrates the demographics of patients, including age, urinary calcium to creatinine ratio, marital status, and comorbidities.

**Table 1:** Demographic and clinical characteristics of the patients (*n* = 50)

Parameter	Number (%) or mean ± SD
Sex	
Male	33 (0.66)
Female	17 (0.34)
Age	52.82 ± 13.25
Urinary calcium to creatinine ratio	0.03 ± 0.01
Currently married	50 (100)
History of drinking	4 (8)
History of smoking	6 (12)
History of urinary tract infection	1 (2)
Body mass index (kg/m^2^)	24.75 ± 2.74
Normal	29 (58)
Overweight	21 (42)
Comorbidities	
Type 2 diabetes mellitus	10 (20)
Hypertension	23 (46)
Temperature (°C)	36.51 ± 0.16
Normal	50 (100)
Fever	0 (0)
Kidney function	
BUA (mmol/L)	344.13 ± 100.41
Normal	38 (76)
High	12 (24)
BUN (mmol/L)	5.84 ± 1.90
Normal	40 (80)
High	10 (20)
SCr (mg/dL)	2.34 ± 0.40
Normal	36 (72)
High	14 (28)
eGFR (mL/min/1.73m^2^)	94.65 ± 31.61
Stage 1	26 (52)
Stage 2	20 (40)
Stage 3	4 (8)

eGFR is expressed in mL/min/1.73 m^2^. Patients were stratified by eGFR into five groups: Stage 1, eGFR > 90; Stage 2, eGFR 60–89; Stage 3, eGFR 30–59; Stage 4, eGFR 15–29; and Stage 5, eGFR < 15

*Abbreviations*: *BUA* Blood uric acid, *BUN* Blood urine nitrogen, *eGFR* Estimated glomerular filtration rate, *SD* Standard deviation

### Sequence-based characterization

A total of 219 samples were collected, and detectable bacterial DNA was found in 47/50 Bladder A samples (urine samples aspirated before bladder disinfection), 44/50 Bladder B samples (newly formed urine after bladder disinfection), 48/50 urine samples of stone kidney pelvis (SKP; the SKP samples were used in our previous study to compare the characteristics of the urinary microbiome of kidney stone patients at different blood pressure stages with that of healthy subjects [21]), 17/19 urine samples of non-stone kidney pelvis (NSKP), and 32/50 blood samples. These samples yielded 11,067,154, raw tags (i.e., raw sequencing reads), 10,767,904 valid tags (i.e., the reads after filtering to remove low-quality reads, reads with adaptors, and reads with unknown bases), and 3232 OTUs. Good’s coverage, which is considered a relative measure of how well the sequences obtained represent the entire population, was 99.92%, indicating genome sequencing was performed at a sufficient depth to identify bacteria in samples.

**Fig. 1:**
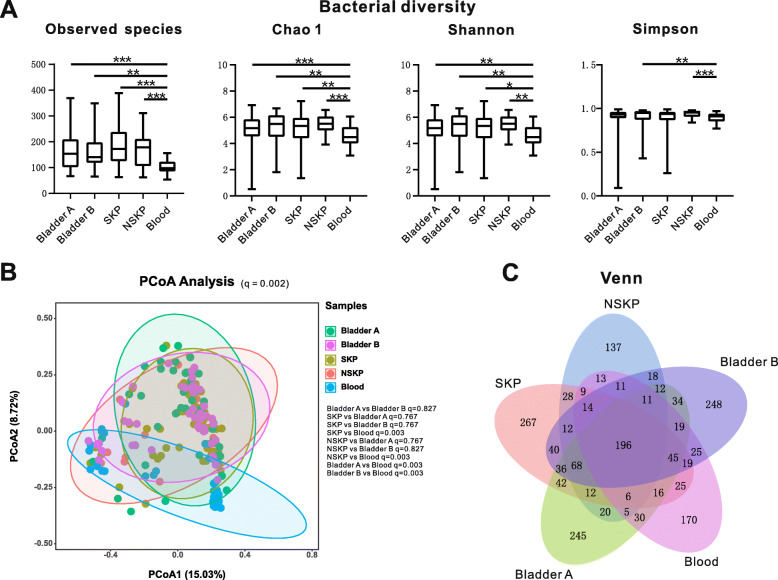
Bacterial diversity and structure among the groups. **a** Comparison of alpha diversity (Observed species, Chao1, Shannon index, and Simpson’s index) between the urinary microbiome of the Bladder A, Bladder B, SKP, NSKP and Blood samples illustrates lower bacterial richness and diversity in Blood samples than the other groups. **b** PCoA shows the bacterial composition clustering of the Bladder A, Bladder B, SKP, NSKP and Blood samples based on Bray–Curtis distances, with each point corresponding to a patient and colored according to the sample type (Bladder A, Bladder B, SKP, NSKP or Blood). PERMANOVA indicated that the bacterial communities between SKP vs Bladder A/Bladder B/ NSKP were non-significantly different (*p* > 0.05), whereas SKP vs Blood was significantly different (*p* = 0.003); Bladder A/Bladder B vs NSKP were non-significantly different (*p* > 0.05), whereas NSKP vs Blood was significantly different (*p* = 0.003); Bladder A vs Bladder B was non-significantly different (*p* > 0.05), whereas Bladder A vs Blood was significantly difference (0.003); Bladder B vs Blood was significantly different (*p* = 0.003). **c** Venn diagram showing that the number of shared OTUs by the KP (including SKP and NSKP) and Blood samples is lower than that shared by the Bladder A/B and KP samples. In addition, the number of shared OTUs by the Bladder A/B and SKP samples is lower than those shared by the Bladder A/B samples and NSKP. Abbreviations: NSKP, non-stone kidney pelvis; OTUs, operational taxonomic units; PCoA, principal coordinates analysis; PERMANOVA, permutational multivariate analysis of variance; SKP: SKP, stone kidney pelvis

To analyse the richness of the urinary microbiome in the Bladder A, Bladder B, SKP, NSKP and blood groups, the number of observed species and the Chao1 estimator were calculated. To assess diversity, the Shannon index and Simpson’s index were calculated (Fig. 1a). The urine samples from Bladder A or Bladder B had similar microbial richness and diversity values to kidney pelvis, including SKP and NSKP (q < 0.05), i.e., the urine samples from the bladder and kidney pelvis had almost an equal number of unique and expected species, unique species and total species, and the relative abundances of these species were equal. In addition, the urine samples had higher Shannon index values than the blood samples (q < 0.05), indicating that the urine samples had more unique species with higher relative abundances.

Based on the permutational multivariate analysis of variance and the principal coordinates analysis (PCoA), we can visualize the similarities of the operational taxonomic units (OTUs) among the samples. Statistically significant differences in the PCoA were only observed between the urine samples of Bladder A/Bladder B/SKP/NSKP and blood samples (q < 0.05; Fig. 1b).

Although the numbers of shared OTUs accounted for 34.76% of the total OTUs in the SKP and Bladder A samples, and the numbers of shared OTUs accounted for 35.45% of the total OTUs in the SKP and Bladder B samples (Fig. 1c), the most common OTUs were present in all of the urine samples (Table S1). In addition, large numbers of OTUs were only present in one sample from each type of urine. For example, 543 OTUs only appeared in one of the SKP samples, including OTU 758, OTU 526 and OTU 2058, etc.; in addition, 819 OTUs were only present once in one of the Bladder A samples, including OTU 3824, OTU 2888, and OTU 3664.

### Bacterial and genus distributions among groups

**Fig. 2:**
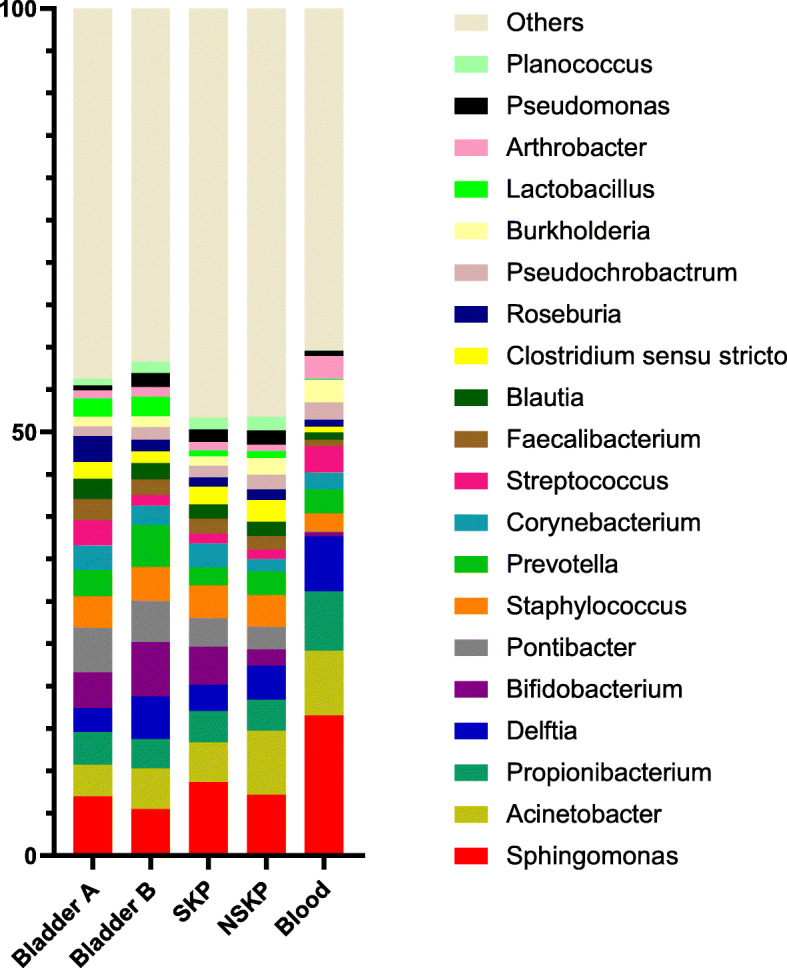
Bacterial genera distribution among the groups. The relative abundances of the major bacterial genera, as determined by 16S rDNA gene sequencing, in the SKP and NSKP samples were more similar to the Bladder A samples than the Blood samples. “Others” includes all detected bacteria. Abbreviations: NSKP, non-stone kidney pelvis; SKP, stone kidney pelvis

The bacterial genera with above 1% of total relative abundance are shown in Fig. 2. The relative abundances of various bacteria were as follows: *Sphingomonas*, *Pontibacter*, and *Bifidobacterium* in the Bladder A group (7.01, 5.33 and 4.21%, respectively), *Bifidobacterium*, *Sphingomonas*, and *Prevotella* in the Bladder B group (6.44, 5.47 and 5.00%, respectively), *Sphingomonas*, *Acinetobacter*, and *Bifidobacterium* in the SKP group (8.67, 4.68 and 4.52%, respectively), and *Acinetobacter, Sphingomonas*, and *Delftia* in the NSKP group (7.55, 7.19 and 4.01%, respectively)*.* Whereas, *Sphingomonas*, *Acinetobacter*, and *Propionibacterium* were predominant in the blood group (16.56, 7.61, and 7.03%, respectively) (Fig. 2).

**Fig. 3:**
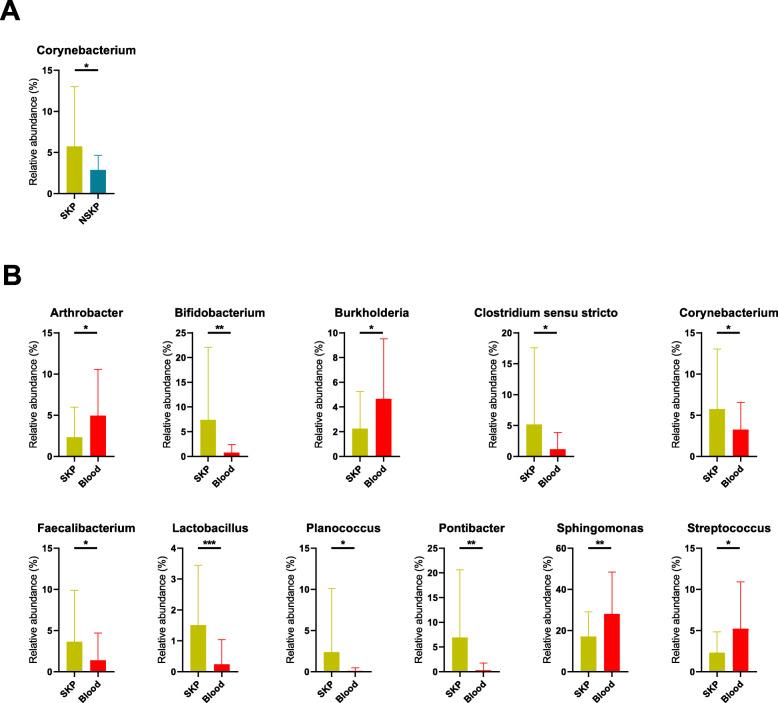
Bacterial abundance showing significant difference between SKP and other samples. White’s non-parametric *t*-test was used to compare the difference of abundance between two groups. *, **, *** means *p* < 0.05, *p* < 0.01, *p* < 0.001, respectively. Abbreviations: NSKP, non-stone kidney pelvis; SKP, stone kidney pelvis

**Fig. 4:**
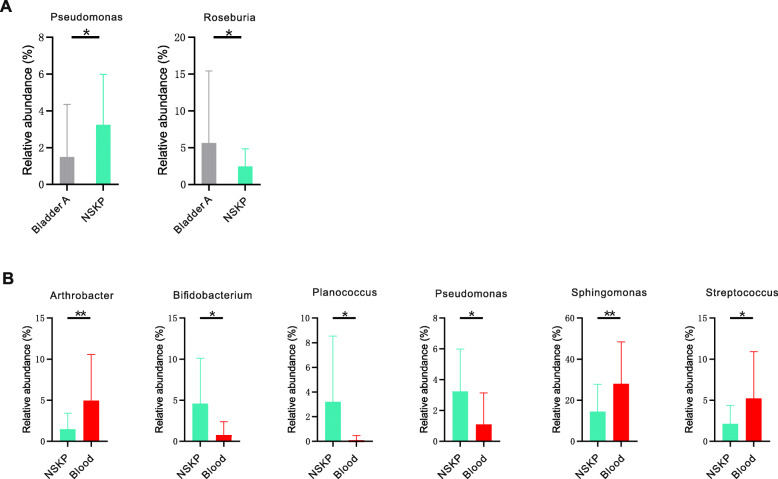
Bacterial abundance showing significant difference between NSKP and other samples. White’s non-parametric *t*-test was used to compare the difference of abundance between two groups. *, **, *** means *p* < 0.05, *p* < 0.01, *p* < 0.001, respectively. Abbreviations: NSKP, non-stone kidney pelvis

The major bacterial genera accounting for more than 1% of the total abundance in the kidney pelvis and other samples were compared. We observed that *Corynebacterium* was enriched in the SKP vs NSKP group (q < 0.05; Fig. 3a). *Pseudomonas* and *Roseburia* were significantly different between the Bladder A and NSKP samples (q < 0.05; Fig. 4a). Interestingly, almost all bacterial genera showing significant differences between the NSKP and blood samples exhibited similar differences between the SKP and blood samples, except for *Pseudomonas*. The abundance of *Lactobacillus* was significantly higher in the SKP sample than in the blood sample (q < 0.05; Fig. 3b and Fig. 4b).

### Bacterial isolation with EQUC and taxa with 16S rRNA sequencing

We observed that some bacteria in the microbial community that were cultured by EQUC could be sequenced by 16S rRNA. For example, *Acetobacteraceae*, *Acidovorax*, *Aerococcus*, *Agrococcus*, *Arthrobacter*, *Brevibacterium*, *Brevundimonas*, *Clostridium*, *Curtobacterium*, *Elizabethkingia*, *Herbaspirillum*, *Janthinobacterium*, *Jeotgalicoccus*, *Kocuria*, *Lactococcus*, *Memnoniella*, *Microbacterium*, *Moraxella*, *Myrmecridium*, *Nigrospora*, *Paenibacillus, Paenisporosarcina*, *Pseudoclavibacter*, *Pseudomonas, Psychrobacter*, *Rhodococcus*, *Roseomonas*, *Saccharopolyspora*, *Sphingomonas* and *Staphylococcus* in the microbial community was detected by both EQUC and 16S rRNA sequencing. However, some of the bacteria in the microbial community that were isolated by EQUC could not be sequenced. For example, most of the samples with isolated *Bacillus* spp., while they were unable to be detected by 16S sequencing (Table S2).

### Associations between the urinary microbiome in the SKP samples and patient characteristics

Pearson correlation analysis was conducted, and we found that the major bacterial genera were not correlated with patient age or the values of BUN and eGFR. Interestingly, PCoA analysis showed that there was no significant difference between the male and female subjects in both samples of Bladder A and SKP (q > 0.05; Figure S1 A and S1 C) or between the pre menstrual and post menstrual females in both Bladder A and SKP samples (q > 0.05; Figure S1 B and S1 D). The 15 most abundant bacterial genera in the subgroups of males and females and pre menstrual and post menstrual females in the Bladder A and SKP subgroups were observed. As shown in Figure S2 A, *Sphingomonas*, *Bifidobacterium*, *Acinetobacter*, *Pontibacter* and *Delftia* were dominant in the male Bladder A samples (6.89, 5.08, 4.66, 4.09 and 3.75%, respectively), whereas *Staphylococcus*, *Pontibacter*, *Sphingomonas*, *Propionibacterium* and *Prevotella* were predominant in the female Bladder A samples (7.75, 7.72, 7.24, 5.84 and 5.66%, respectively). The pre menstrual and post menstrual female Bladder A samples were dominated by *Pontibacter*, *Streptococcus*, *Sphingomonas*, *Propionibacterium* and *Bifidobacterium* (15.36, 11.27, 7.49, 6.59 and 3.32%, respectively); the post-menstrual samples were dominated by *Staphylococcus*, *Prevotella*, *Sphingomonas*, *Roseburia* and *Propionibacterium* (11.26, 8.22, 7.10, 5.57 and 5.39%, respectively; Figure S2 B). Additionally, the Bladder A samples did not exhibit significant differences between male and female samples or between pre menstrual and post menstrual females (q > 0.05). The samples of males in the SKP group were dominated by *Sphingomonas* (8.52%), *Bifidobacterium* (5.30%), *Acinetobacter* (4.83%), *Propionibacterium* (3.28%) and *Delftia* (3.16%). (Figure S2 C), while the samples of the female subjects were dominated by *Sphingomonas* (8.95%), *Staphylococcus* (8.25%), *Pontibacter* (4.89%), *Propionibacterium* (4.42%) and *Acinetobacter* (4.40%), etc. When the female SKP samples were divided into pre menstrual and post menstrual subgroups, the samples of pre menstrual females were dominated by *Staphylococcus*, *Pontibacter*, *Sphingomonas*, *Faecalibacterium* and *Bacteroides* (13.85, 12.36, 6.54, 5.34 and 4.19%, respectively), whereas the samples of post menstrual females were dominated by *Sphingomonas*, *Acinetobacter*, *Staphylococcus*, *Propionibacterium* and *Delftia* (10.26, 6.33, 5.20, 4.71 and 4.68%, respectively, Figure S2 D). The 15 most abundant bacterial genera were compared between subgroups of males and females and between pre menstrual and post menstrual females, and there was no significant difference (q > 0.05).

Notably, several bacterial genera showed associations with patient characteristics. For example, *Lactobacillus* was positively correlated with fasting blood glucose (*r* = 0.333, *p* = 0.021), *Arthrobacter* was positively correlated with BMI (*r* = 0.551, *p* < 0.001), *Prevotella* was negatively correlated with BMI (*r* = − 0.369, *p* = 0.010).

## Discussion

The old dogma that urine in healthy bladder is sterile has been challenged with the application of 16S rRNA sequencing technology and the EQUC technique [22–24]. The role of the bladder urinary microbiome in urinary tract disease has recently been revealed using catheterized urine samples [11, 22, 24, 25]. Similarly, an increasing number of studies are exploring the notion that bacteria in human blood do not necessarily equate to infection [15, 16, 26, 27]. The present study explored the existence of the urinary microbiome in the kidney pelvis and its association with the microbiome in the bladder and blood in patients with kidney stones. In the meantime, the microbiome composition of kidney pelvis with and without stones was compared. For the first time, repeated disinfection of the bladder under cystoscopy was used to eliminate the influence of the organisms in the bladder. This was followed by the use of EQUC, a technique for culturing living urinary bacteria [23], to verify the effect of disinfection. Importantly, we concluded that although the urinary microbiome composition in SKP was affected by the clinical parameters of several patients, the influencing factors are not similar to the human gut microbiome.

The numbers of sequencing-positive Bladder A, Bladder B, SKP and NSKP samples were similar; however, this number was lower in the blood group. Consistent with the sequencing results, the comparison of alpha diversity revealed that the blood samples exhibited the lowest bacterial diversity compared with the urine samples either from the kidney pelvis or bladder. These findings suggest that the bacterial biomass in the blood is lower than that in the urine of patients with kidney stones. This may be attributed to the kidney function insufficiency noted in approximately half of the patients. A recent study reported that patients with chronic kidney disease had decreased bacterial diversity in their blood [16].

Both PCoA and Venn diagram demonstrated that the microbiome composition in the kidney pelvis urine was similar to that in the bladder urine. The resemblance between the urinary microbiome in the kidney pelvis and bladder was consistent with the findings of Dornbier et al., in which the microbiomes of the urine in the upper urinary tract and the bladder of patients with kidney stones were similar [13]. In the present study, the original urine in the bladder was removed, followed by repeated disinfection of the bladder with iodophor and washing with normal saline. However, Dornbier et al. did not disinfect the bladder prior to collecting kidney pelvis urine. Thus, the findings in their and our study suggest that bladder disinfection might not be necessary for collecting kidney pelvis urine samples or that bladder urine can be used in studies describing the characteristics of kidney pelvis urine.

Some bacterial genera in the bladder and kidney pelvis, including *Acinetobacter*, *Bifidobacterium*, *Corynebacterium*, *Lactobacillus*, *Staphylococcus*, and *Streptococcus*, were also previously reported as the dominant bacteria in patients with urinary stones [13, 28]. Although the major bacteria in the present study, including *Delftia, Propionibacterium* and *Sphingomonas*, have not been reported in a previous study on the urinary microbiome in kidney stone patients, they have been demonstrated to be associated with human health status in the urine microbiome. For example, *Delftia* was overrepresented in urine from patients with type 2 diabetes mellitus [29], *Propionibacterium* was enriched in the urine of patients with bacterial vaginosis [10], and *Sphingomonas* tended to be more abundant in kidney transplantation individuals with stable status [30]. More studies are needed to explore the roles of these bacteria in the human urinary microbiome.

Most of the major bacterial genera in SKP and NSKP were similar. However, *Corynebacterium* was significantly increased in the SKP group compared with the NSKP group. Previous studies have confirmed that *Corynebacterium* spp. is associated with stone formation [31, 32]. Similarly, *Corynebacterium* was considered a major bacterial genus in two samples in the report of Dornbier et al. on the microbiome in human kidney stones [13]. Thus, the increased level of *Corynebacterium* might be a protective response in the SKP in KSD patients.

We observed that there was no significant difference in the major bacterial genera between Bladder A and SKP samples, while the abundances of *Pseudomonas* and *Roseburia* were significantly higher or lower in NSKP compared to Bladder A, respectively. These findings might be because vesicoureteral reflux is common in patients with kidney stones [33], i.e., the possibility of bladder urine flowing back to the kidney pelvis increases, which may result in increased levels of shared microorganisms between SKP and bladder urine.

When the microbiomes in the kidney pelvis and blood were compared, we found that the numbers of the major bacterial genera showing significant differences between the SKP and blood samples were greater than those between the NSKP and blood samples. These findings indirectly suggest that the occurrence of kidney stones affected the urinary microbiome in the kidney pelvis. It is worth noting that the abundance of *Lactobacillus* was significantly higher in SKP compared to blood but not in NSKP compared to blood. This finding indicates that the occurrence of kidney stones may activate the self-protective response in the kidney pelvis urinary microbiome, since *Lactobacillus* is a probiotic that is able to prevent kidney stones because of its oxalate-degrading activities [34].

We observed that the EQUC results for some samples were not consistent with the results of detected taxonomy (bacterial genus) in bladder urine. Similar findings were reported by Dornbier RA et al.; for example, one female sample contained *Ochrobactrum* spp. based on EQUC, but the sample was dominated by *Corynebacterium* and Enterobacteriaceae [13]. These findings suggest that it is beneficial for researchers and clinicians to obtain a comprehensive understanding of the urinary microbiome when using both 16S rRNA sequencing of the microbiome and bacterial isolates with EQUC.

Although our present study did not reveal that patient gender, age or menstrual status were correlated with bacterial composition in the bladder and SKP urine at the present sample size, which is dissimilar to the human gut microbiome, we found that several major bacterial genera were associated with patient clinical parameters. For example, the abundance of *Lactobacillus* increased with patient fasting blood glucose, which is similar to a previous human urinary microbiome study conducted by Chen JW et al. In their study, *Lactobacillus* was overrepresented in the high haemoglobin A1c group [29]. In addition, we found that a high abundance of *Prevotella* was linked to a low BMI. It is difficult to determine the role of *Prevotella* in human health, since a recent study reported that the correlations between *Prevotella* and BMI in previous human studies were not consistent [35].

## Conclusions

This study demonstrated the existence of the urinary microbiome in the kidney pelvis after bladder disinfection, and the urinary microbiome in the kidney pelvis was similar to that in the bladder urine, but the microbiomes in both bladder urine and kidney pelvis urine were dissimilar to the blood microbiome. To some extent, we revealed that the urinary microbiome in the SKP samples was different from that in the NSKP samples, which is related to alterations in *Corynebacterium* in the SKP samples. Although the mechanism responsible for this observation remain unclear, the results suggest that kidney stones can be improved by targeting modification of *Corynebacterium*; for example, dietary therapy. In addition, we found that the bacterial profile in SKP was related to the clinical characteristics of the patients, indicating that modifying these characteristics, including fasting blood glucose and BMI, using microbiome-based therapy might play a role in controlling kidney stone occurrence. It is worth noting that the present study demonstrated that the presence of bacteria in the urine bladder and kidney pelvis, and the blood. Further study should characterize the consistency of their alterations, which might provide strategies for holistic therapy in maintaining microbiome balance.

A limitation of this analysis is that the sample size was insufficient, which reduced the statistical power of detecting true differences in this study. Moreover, although the bladder was repeatedly disinfected prior to the collection of kidney pelvis urine, this cannot completely prevent contamination of the kidney pelvis urine by the bladder urine. As the cystoscope and the tube were passed through the urethra and bladder into the kidney pelvis to collect urine samples; it was possible for intracellular bacterial colonization in the urothelium to contaminate the instruments and samples [36]. Therefore, in the future, it will be necessary to collect samples using percutaneous kidney puncture to verify whether the kidney pelvis urine is contaminated by bladder urine. Third, we excluded patients with negative Bladder A urine cultures; however, bacteria may be present in the renal pelvis urine of these patients. This is a selective bias, which can be improved by using percutaneous kidney puncture to collect pelvis urine in the future. Fourth, not having renal pelvis samples from individuals without kidney stones to compare the urine microbiome in stone formers and non-stone formers is one of limitation of the present study. Last, only PCR products of 9 samples were randomly selected to be sequenced twice because of limited financial support of our project, which might skew the results of the present study.

## Methods

### Patient selection and sample size

The study protocol was approved by the Affiliated Wuxi Second Hospital, Nanjing Medical University, Ethical Review Committee (ref. 201,802), and patients provided written informed consent for the use of their samples. The process was in compliance with the Declaration of Helsinki, which included risk and benefit assessment. Kidney calcium stones were confirmed by abdominal X-ray, ultrasonography, and computed tomography scans. Patients undergoing ureteroscopic lithotripsy were recruited between October 2018 and April 2019. The following exclusion criteria were applied: menstruation or pregnancy, cancer, autoimmune disease, urinary tract disease (including urethritis, prostatitis, benign prostatic hyperplasia, kidney cyst, and cystitis), urinary tract deformity, known urinary tract infection based on clinical assessment, urinary catheterization in the previous 4 weeks, and treatment with antibiotics in the previous 4 weeks. Based on previous studies that compared microbiomes in different body niches [37, 38], 50 participants were sufficient to determine the differences in their microbiomes.

### Specimen collection

Six surgeons with ≥10 years of experience in conducting ureteroscopic lithotripsy were trained to use the procedures of disinfection and collection of urine samples; this guaranteed that the surgeons used unified methods to disinfect the urethra and its surrounding tissues and bladder. In addition, they were required to use unified methods to insert the ureteroscope and tube into the kidney pelvis. Specifically, after the administration of general anaesthesia to the patient, the following procedures were used to collect samples: (a) Disinfection of the perineum area and the urinary meatus with iodophor at least three times. (b) Insertion of a ureteroscope (Richard Wolf, Knittligen, Germany) and withdrawal of 3 mL of urine from the bladder (labelled Bladder A). EQUC technique was used to detect the presence of living bacteria (> 10 colony-forming units per mL) in bladder urine [23]. Patients with negative culture results were excluded since previous studies demonstrated that there are living bacteria in human bladders using EQUC [22, 39]. (c) The residual urine was aspirated from the bladder. The bladder was disinfected with iodophor three times, and the ureteroscope was used to check whether the bladder was completely full of iodophor. The iodophor was maintained in the bladder for ≥30 s prior to aspiration. Subsequently, 3 mL of iodophor from the last lavage was aspirated, and the EQUC technique was also applied again to verify the presence of detectable bacteria in the newly formed urine after disinfection [22]. If the bacteria in the bladder were not completely killed, they could contaminate the urine samples in the kidney pelvis, since kidney pelvis urine samples were collected by cystoscopy via the bladder. Thus, patients with positive culture results after bladder disinfection were excluded from the study. (d) Sterile normal saline was used to lavage the bladder three times and promote discharge of iodophor. A 6–7F tube (New District HuaSheng Medical Instrument Co., Ltd., Suzhou, China) was placed into the ureteroscope, and both were gently inserted into the kidney pelvis with stone(s). Urine (3 mL) was aspirated from the kidney pelvis, and the samples were labelled SKP (urine of stone kidney pelvis). The ureteroscope and the tube were withdrawn. (e) Procedure c was then repeated. Subsequently, a new ureteroscope was used, and another 6–7F tube was inserted into the ureteroscope. Both the ureteroscope and the tube were inserted into the other kidney pelvis without stone(s) of the same patients who provided the SKP sample, and urine (3 mL) was aspirated. The sample was labelled NSKP. NSKP samples were not collected from patients in whom the ureter was too narrow to insert the ureteroscope. (f) Newly formed urine (3 mL) in the bladder was collected and labelled Bladder B.

### Urine culture

The procedure of EQUC was as follows: 0.1 mL of urine was inoculated onto 5% sheep blood agar plate (BAP), chocolate and colistin, and nalidixic acid (CNA) agars, streaked for quantitation, and incubated in 5% CO_2_ at 35 °C for 48 h. For a second set of BAPs, each was inoculated with 0.1 ml of urine and incubated in room atmosphere at 35 °C and 30 °C for 48 h. Next, 0.1 ml of urine was inoculated onto each of two CDC anaerobic 5% sheep blood agar plates and incubated in a Campy gas mixture (5% O_2_, 10% CO_2_, 85% N) at 35 °C for 48 h. The detection level was 10 CFU/ml, which was represented by 1 colony on any of the plates. Finally, to detect any bacterial species that may be present at quantities lower than 10 CFU/ml, 1.0 ml of urine was placed in thioglycolate medium (BD BBL prepared tubed media) and incubated aerobically at 35 °C for 5 days. If growth was visible in the thioglycolate medium, the medium was mixed, and a few drops were plated on BAP and CDC anaerobic 5% sheep blood agar plates for isolation and incubated aerobically and anaerobically at 35 °C for 48 h. All of the plates and media used in the present study were prepared by Comagal Microbial Technology Co., Ltd., China. All procedures of EQUC, bacterial isolation and genomic DNA isolation were performed in a biosafety cabinet that was sterilized using chlorine disinfectant, alcohol solution and ultraviolet irradiation in advance. PCR for the amplification of the 16S rRNA gene was carried out using universal primers; 27F as the forward primer, and 1492 as the reverse primer. The PCR band from each of the isolates was Sanger sequenced, and the bacterium was identified to the strain level using BLAST. If the genome sequence of a bacterium was identified as heterozygous three times or was difficult to bind, the bacterium was defined as an unidentified bacterium. The similarity of the bacterial sequence cutoff is 97% [40]. When the similarity of several bacteria was above 97%, only the one with the highest similarity was displayed.

An experienced nurse collected all blood samples as follows: (a) The skin of the intravenous injection site was disinfected with iodophor; (b) A needle was inserted into the vessel, and blood (5 mL) was drawn; (c) The stopper of a vacutainer containing ethylenediaminetetraacetic acid (EDTA) was removed, the blood was gently injected into the vacutainer, and the stopper was then put back on the vacutainer.

### Specimen processing and sequencing

The Bladder A, Bladder B, SKP, and NSKP samples were immediately placed in a foam box with ice packages in the operation room and then immediately transferred to the lab in our hospital within 10 mins. A total of 1 mL of urine was centrifuged at 20,000×g for 30 min, and the resulting pellet was resuspended in 150 μL of lysis buffer (BGI Inc., Shenzhen, China). As described in our previous study, Sera-Mag™ SpeedBeads Carboxylate-Modified Magnetic Particles (GE Healthcare UK Ltd., Buckinghamshire, UK) were used to extract the DNA according to the instructions provided by the manufacturer [21]. Buffy coat specimens were used for the isolation of blood bacterial DNA [16], and the same method as that used for the collection of the urine samples was applied. The DNA concentration was detected using a Qubit Fluorometer.

Bacterial DNA was amplified via polymerase chain reaction with 35 cycles using the universal primers 341F and 806R [41, 42], which target the variable V3-V4 regions of the 16S rRNA gene. Amplicons were analysed via gel electrophoresis and purified using a QIAquick gel extraction kit (QIAGEN, Hilden, Germany). Products were diluted to 10 ng/μL, and 5 μL of each sample was pooled for PE300 sequencing using a HiSeq 2500 system (Illumina Inc., San Diego, CA, USA). DNA extraction negative controls with normal saline, but no urine or blood, were added to assess the contribution of extraneous DNA from reagents. Moreover, negative controls without template DNA (i.e., no PCR product) were added to be sequenced.

### Bioinformatic analysis

Paired-end reads were assigned to the samples based on their unique barcode and truncated by cutting off the barcode and primer sequence. Paired-end reads were merged using FLASH. Quality filtering of the raw tags was performed under specific filtering conditions to obtain high-quality clean tags according to fqtrim v0.94. Chimeric sequences were filtered, and sequences with ≥97% similarity were assigned to the same OTUs using Vsearch v2.3.4. Representative sequences were chosen for each OTU, and taxonomic data were then assigned to each representative sequence using the Ribosomal Database Project (RDP) 11.5, released on September 30, 2016, with a confidence value of 0.8 as the cutoff. The sequences were used as queries to search against the National Center of Biotechnology Information (NCBI) nucleotide database (downloaded in April 2019). Samples with < 30,000 clean tags were removed. Contaminant sequences (based on the negative controls) were removed using Decontam v1.2.1 and the threshold *p* < 0.1 [43, 44].

The OTU abundance data were normalized using a standard sequence number corresponding to the sample with the smallest number of sequences. Alpha diversity was used to analyse the complexity of species diversity in each sample using QIIME v1.8.0 to calculate the alpha-diversity index, including observed species, Chao1, Shannon index, and Simpson’s index [45]. The observed species is a count of the number of unique species that occur in a sample or community. Chao 1 is a measurement of the species expected in samples given all bacterial species that are identified in the samples [46]. The Shannon index is the number of unique species and their relative abundances within a sample. The Simpson index evaluates the relative abundances of all species in a community [45]. Beta diversity analysis was used to evaluate differences in species complexity between different types of samples [47]. To identify similarities among the SKP, NSKP, Bladder A, Bladder B, and blood samples, the samples were compared using a PCoA with Bray–Curtis dissimilarity at the OTU level (R package vegan) [47]. Permutational multivariate analysis of variance was applied to determine whether the differences in the bacterial communities of the groups were significant. Based on the OTU abundance, a Venn diagram was used to display the number of microbial OTUs shared by the five groups of samples. Patient characteristics were collected by medical chart review.

### Kidney function indicators

The kidney function estimators were measured on an Olympus AU5421–04 (Beckman Olympus, USA) in the clinical lab in our hospital. The normal range of blood urea nitrogen (BUN) was defined as 2.5–7.1 mmol/L, and blood urea acid (BUA) was 200–430 mmol/L in men and 140–360 mmol/L in women. eGFR was expressed in ml/min/1.73 m^2^. Patients were stratified into five groups by baseline eGFR: Stage 1, eGFR > 90; Stage 2, eGFR 60–89; Stage 3, eGFR 30–59; Stage 4, eGFR 15–29; and Stage 5, eGFR < 15.

### Statistical analysis

The data were not normally distributed. Thus, for pairwise comparisons of bacterial communities, White’s nonparametric *t*-test was used [48]. For group comparisons of alpha indices, the Wilcoxon signed-rank test was used. In addition, the correlation of the bacterial genera in the SKP samples and patient characteristics was analysed using Pearson correlation analysis. All *P*-values were corrected using the Benjamin–Hochberg false discovery rate correction, and q-values < 0.05 were considered statistically significant [48].

## Supplementary information

**Additional file 1: Table S1.** The 100 most common OTUs in the five groups of samples. Abbreviation: operational taxonomic units, OTUs.

**Additional file 2: Table S2.** Bacteria in bladder urine detected by EQUC and 16S rRNA sequencing Abbreviation: colony forming units, CFU. ^a^ The similarity of bacterial sequence cutoff is 97%. ^b^ If the genome sequence of a bacterium was identified as heterozygous three times or was difficult to bind, the bacterium was defined as an unidentified bacterium.

**Additional file 3: Figure S1.** Bacterial structure between groups of males and females and pre menstrual and post menstrual females**.** PCoA shows the bacterial composition clustering of the groups based on Bray–Curtis distances, with each point corresponding to a patient and coloured according to the sample type of male or female in the SKP samples **(A)**, pre menstrual and post menstrual females in the SKP samples **(B)**, male or female in Bladder A samples **(C)**, and pre menstrual and post menstrual females in the Bladder A samples **(D)**. PERMANOVA indicated that the bacterial communities between males and females were not significantly different in the SKP samples (*p* > 0.05).

**Additional file 4: Figure S2.** Bacterial genera distribution between the groups. The relative abundances of the major genera in males and females in the SKP samples **(A)**, in pre menstrual and post menstrual females in the SKP samples **(B)**, in males and females in the Bladder A samples **(C)** and in pre menstrual and post menstrual females in the Bladder A samples **(D)**, as determined by 16S rDNA gene sequencing, in the SKP and NSKP samples were more similar to the Bladder A samples than the blood samples. “Others” includes all detected bacteria.

## Data Availability

The sequencing data from this study were deposited in the GenBank Sequence Read Archive under accession number SRP218817 (https://www.ncbi.nlm.nih.gov/bioproject/PRJNA561017/).

## Abbreviations

SKP: Stone kidney pelvis
BUA: Blood uric acid
eGFR: Estimated glomerular filtration rate
EQUC: Expanded quantitative urine culture
NSKP: Non-stone kidney pelvis
NSKP: Non-stone kidney pelvis
OTUs: Operational taxonomic units
PCoA: Principal coordinates analysis
SKP: Stone kidney pelvis
