# Supplementary material for: The pelvis urinary microbiome in patients with kidney stones and clinical associations
Source: BMC Microbiol. 2020 Nov 5;20:336. doi: 10.1186/s12866-020-01992-4 (PMC7643416; doi:10.1186/s12866-020-01992-4)
Supplement: Supplementary file 1 — Additional file 1: Table S1. The 100 most common OTUs in the five groups of samples. Abbreviation: operational taxonomic units, OTUs. [file 12866_2020_1992_MOESM1_ESM.doc]

**Table S1 The 100 most common OTUs in the five groups of samples**

| OTU ID | Taxon | SKP | NSKP | Bladder A | Bladder B | Blood |
| --- | --- | --- | --- | --- | --- | --- |
| OTU100 | Lachnospiraceae | 28/48 | 13/17 | 36/47 | 27/44 | 8/32 |
| OTU102 | Bacteroides | 34/48 | 13/17 | 35/47 | 36/44 | 8/32 |
| OTU104 | Pseudomonas veronii | 36/48 | 14/17 | 35/47 | 32/44 | 19/32 |
| OTU107 | Chryseobacterium | 18/48 | 8/17 | 22/47 | 25/44 | 5/32 |
| OTU11 | Acinetobacter lwoffii | 36/48 | 15/17 | 26/47 | 26/44 | 5/32 |
| OTU110 | Turicibacter | 19/48 | 8/17 | 24/47 | 22/44 | 3/32 |
| OTU1108 | Acinetobacter | 15/48 | 7/17 | 18/47 | 18/44 | 7/32 |
| OTU111 | Acinetobacter guillouiae | 27/48 | 9/17 | 27/47 | 28/44 | 9/32 |
| OTU115 | Bacteroides | 17/48 | 7/17 | 23/47 | 19/44 | 5/32 |
| OTU116 | Caulobacteraceae | 19/48 | 6/17 | 11/47 | 16/44 | 12/32 |
| OTU1217 | Bifidobacterium | 25/48 | 9/17 | 25/47 | 24/44 | 1/32 |
| OTU1232 | Propionibacterium acnes | 40/48 | 12/17 | 32/47 | 35/44 | 17/32 |
| OTU124 | Lachnospiraceae | 19/48 | 7/17 | 22/47 | 21/44 | 2/32 |
| OTU128 | Ruminococcus gnavus | 25/48 | 7/17 | 24/47 | 16/44 | 7/32 |
| OTU129 | Mitochondria | 33/48 | 9/17 | 18/47 | 16/44 | 28/32 |
| OTU135 | Bradyrhizobium | 28/48 | 10/17 | 31/47 | 27/44 | 17/32 |
| OTU136 | Bacillus | 19/48 | 8/17 | 18/47 | 21/44 | 0/32 |
| OTU14 | Acinetobacter johnsonii | 42/48 | 17/17 | 42/47 | 44/44 | 13/32 |
| OTU148 | Gemmiger formicilis | 24/48 | 10/17 | 28/47 | 25/44 | 3/32 |
| OTU1579 | Streptophyta | 38/48 | 14/17 | 39/47 | 30/44 | 7/32 |
| OTU161 | Agrobacterium | 17/48 | 9/17 | 18/47 | 14/44 | 7/32 |
| OTU166 | Peptostreptococcaceae | 18/48 | 9/17 | 23/47 | 19/44 | 4/32 |
| OTU1711 | Agrobacterium | 17/48 | 9/17 | 15/47 | 15/44 | 13/32 |
| OTU172 | Blautia | 37/48 | 14/17 | 38/47 | 37/44 | 9/32 |
| OTU177 | Novosphingobium | 29/48 | 9/17 | 29/47 | 26/44 | 12/32 |
| OTU178 | Methylobacterium adhaesivum | 17/48 | 7/17 | 17/47 | 21/44 | 5/32 |
| OTU18 | Pseudomonas fragi | 27/48 | 10/17 | 20/47 | 26/44 | 10/32 |
| OTU19 | Prevotella copri | 40/48 | 17/17 | 38/47 | 36/44 | 13/32 |
| OTU192 | Roseburia | 24/48 | 9/17 | 30/47 | 20/44 | 8/32 |
| OTU2 | Streptophyta | 39/48 | 11/17 | 43/47 | 37/44 | 10/32 |
| OTU219 | Coprococcus eutactus | 14/48 | 7/17 | 23/47 | 17/44 | 2/32 |
| OTU222 | Corynebacterium | 26/48 | 7/17 | 26/47 | 22/44 | 12/32 |
| OTU229 | Psychrobacter | 15/48 | 6/17 | 22/47 | 20/44 | 6/32 |
| OTU23 | Lactobacillus helveticus | 26/48 | 6/17 | 26/47 | 23/44 | 1/32 |
| OTU236 | Mitochondria | 24/48 | 7/17 | 21/47 | 20/44 | 2/32 |
| OTU24 | Clostridiaceae | 26/48 | 13/17 | 28/47 | 20/44 | 8/32 |
| OTU247 | Veillonella dispar | 18/48 | 4/17 | 21/47 | 19/44 | 9/32 |
| OTU249 | Coprococcus | 25/48 | 9/17 | 27/47 | 22/44 | 4/32 |
| OTU254 | Ruminococcus | 30/48 | 12/17 | 31/47 | 24/44 | 5/32 |
| OTU2579 | Comamonadaceae | 19/48 | 7/17 | 17/47 | 17/44 | 11/32 |
| OTU258 | Caulobacteraceae | 33/48 | 9/17 | 25/47 | 26/44 | 11/32 |
| OTU261 | Psychrobacter | 23/48 | 7/17 | 19/47 | 23/44 | 7/32 |
| OTU2662 | Prevotella copri | 33/48 | 13/17 | 30/47 | 31/44 | 13/32 |
| OTU268 | Coprococcus | 25/48 | 10/17 | 31/47 | 21/44 | 7/32 |
| OTU27 | Bacteroides ovatus | 18/48 | 9/17 | 28/47 | 17/44 | 5/32 |
| OTU272 | Corynebacterium | 19/48 | 4/17 | 18/47 | 20/44 | 1/32 |
| OTU2975 | Sphingomonas | 24/48 | 9/17 | 20/47 | 25/44 | 14/32 |
| OTU3 | Staphylococcus epidermidis | 45/48 | 12/17 | 46/47 | 43/44 | 26/32 |
| OTU31 | Paracoccus | 34/48 | 13/17 | 28/47 | 31/44 | 8/32 |
| OTU3187 | Corynebacterium lubricantis | 37/48 | 9/17 | 41/47 | 39/44 | 15/32 |
| OTU32 | Cellulosimicrobium | 21/48 | 8/17 | 21/47 | 14/44 | 4/32 |
| OTU3257 | Arthrobacter woluwensis | 32/48 | 10/17 | 33/47 | 30/44 | 16/32 |
| OTU3372 | Brucellaceae | 22/48 | 9/17 | 16/47 | 14/44 | 14/32 |
| OTU3421 | Streptophyta | 30/48 | 9/17 | 29/47 | 25/44 | 7/32 |
| OTU3499 | Bifidobacterium adolescentis | 41/48 | 14/17 | 35/47 | 37/44 | 6/32 |
| OTU350 | Eubacterium biforme | 18/48 | 9/17 | 21/47 | 19/44 | 4/32 |
| OTU3522 | Streptococcus | 29/48 | 7/17 | 25/47 | 23/44 | 9/32 |
| OTU37 | Roseburia faecis | 37/48 | 13/17 | 41/47 | 39/44 | 8/32 |
| OTU38 | Sphingobium | 15/48 | 10/17 | 20/47 | 18/44 | 16/32 |
| OTU39 | Streptococcus alactolyticus | 31/48 | 9/17 | 27/47 | 30/44 | 12/32 |
| OTU40 | Enhydrobacter | 43/48 | 13/17 | 42/47 | 39/44 | 17/32 |
| OTU4013 | Bifidobacterium | 32/48 | 11/17 | 33/47 | 30/44 | 8/32 |
| OTU4051 | Bifidobacterium bifidum | 12/48 | 6/17 | 23/47 | 23/44 | 2/32 |
| OTU4124 | Prevotella copri | 19/48 | 10/17 | 20/47 | 22/44 | 7/32 |
| OTU421 | Ruminococcus gnavus | 25/48 | 10/17 | 26/47 | 22/44 | 4/32 |
| OTU43 | Corynebacterium | 21/48 | 7/17 | 23/47 | 21/44 | 5/32 |
| OTU44 | Corynebacterium | 41/48 | 9/17 | 44/47 | 39/44 | 17/32 |
| OTU45 | Faecalibacterium prausnitzii | 38/48 | 12/17 | 44/47 | 38/44 | 11/32 |
| OTU476 | Erysipelotrichaceae | 23/48 | 12/17 | 23/47 | 18/44 | 2/32 |
| OTU48 | Streptococcus infantis | 39/48 | 11/17 | 37/47 | 39/44 | 17/32 |
| OTU49 | Cloacibacterium | 27/48 | 10/17 | 29/47 | 27/44 | 3/32 |
| OTU5 | Escherichia coli | 34/48 | 10/17 | 29/47 | 28/44 | 7/32 |
| OTU50 | Deinococcus geothermalis | 20/48 | 6/17 | 25/47 | 19/44 | 1/32 |
| OTU503 | Dorea | 16/48 | 6/17 | 21/47 | 18/44 | 7/32 |
| OTU51 | Rhodococcus | 16/48 | 8/17 | 19/47 | 17/44 | 5/32 |
| OTU513 | Agrobacterium | 14/48 | 7/17 | 16/47 | 20/44 | 7/32 |
| OTU53 | Bifidobacterium animalis | 19/48 | 6/17 | 20/47 | 19/44 | 1/32 |
| OTU54 | Lactobacillus reuteri | 23/48 | 9/17 | 25/47 | 20/44 | 0/32 |
| OTU59 | Megamonas | 29/48 | 10/17 | 24/47 | 30/44 | 7/32 |
| OTU6 | Streptophyta | 42/48 | 15/17 | 42/47 | 38/44 | 10/32 |
| OTU60 | Serratia marcescens | 18/48 | 7/17 | 17/47 | 14/44 | 12/32 |
| OTU64 | Planococcus | 27/48 | 11/17 | 25/47 | 27/44 | 1/32 |
| OTU66 | Burkholderia | 48/48 | 17/17 | 46/47 | 44/44 | 28/32 |
| OTU68 | Enterococcus casseliflavus | 21/48 | 7/17 | 24/47 | 21/44 | 2/32 |
| OTU69 | Peptostreptococcaceae | 26/48 | 13/17 | 32/47 | 28/44 | 7/32 |
| OTU7 | Bifidobacterium longum | 41/48 | 13/17 | 38/47 | 36/44 | 7/32 |
| OTU70 | Ochrobactrum | 24/48 | 12/17 | 24/47 | 22/44 | 12/32 |
| OTU72 | Bacteroides plebeius | 19/48 | 9/17 | 28/47 | 18/44 | 8/32 |
| OTU76 | Ruminococcus bromii | 34/48 | 11/17 | 35/47 | 30/44 | 8/32 |
| OTU77 | Gemmiger formicilis | 25/48 | 8/17 | 24/47 | 25/44 | 5/32 |
| OTU8 | Delftia | 16/48 | 10/17 | 17/47 | 15/44 | 12/32 |
| OTU81 | Pseudomonas alcaligenes | 20/48 | 8/17 | 20/47 | 13/44 | 10/32 |
| OTU849 | Blautia | 32/48 | 13/17 | 33/47 | 28/44 | 6/32 |
| OTU9 | Sphingomonas | 48/48 | 17/17 | 47/47 | 44/44 | 28/32 |
| OTU90 | Stenotrophomonas geniculata | 19/48 | 9/17 | 15/47 | 19/44 | 11/32 |
| OTU92 | Bacteroides uniformis | 20/48 | 9/17 | 25/47 | 19/44 | 4/32 |
| OTU921 | Pseudomonas | 22/48 | 6/17 | 19/47 | 23/44 | 7/32 |
| OTU93 | Blautia | 33/48 | 14/17 | 39/47 | 36/44 | 8/32 |
| OTU96 | Arthrobacter | 38/48 | 11/17 | 34/47 | 31/44 | 17/32 |
| OTU99 | Knoellia subterranea | 23/48 | 9/17 | 22/47 | 22/44 | 7/32 |

Abbreviation: operational taxonomic units, OTU
