# Supplementary material for: The pelvis urinary microbiome in patients with kidney stones and clinical associations
Source: BMC Microbiol. 2020 Nov 5;20:336. doi: 10.1186/s12866-020-01992-4 (PMC7643416; doi:10.1186/s12866-020-01992-4)
Supplement: Supplementary file 2 — Additional file 2: Table S2. Bacteria in bladder urine detected by EQUC and 16S rRNA sequencing Abbreviation: colony forming units, CFU. a The similarity of bacterial sequence cutoff is 97%. b If the genome sequence of a bacterium was identified as heterozygous three times or was difficult to bind, the bacterium was defined as an unidentified bacterium. [file 12866_2020_1992_MOESM2_ESM.doc]

**Table S2 Bacteria in bladder urine detected by EQUC and 16S rRNA sequencing**

| **Subject ID** | **EQUC** | | | **16S rRNA sequencing** | | |
| --- | --- | --- | --- | --- | --- | --- |
|  | **Isolated bacteria** | **Similarity (%)** | **CFU/ml** | **Taxa** |  | **Relative abundance (%)** |
| 1 | Bacillus sp. E17 | 99.85 | 10 | Bacillus |  | 0 |
|  | Staphylococcus saprophyticus strain 3-16 | 99.93 | 10 | Staphylococcus |  | 1.75 |
| 2 | Bacillus aerius strain UB02 | 100 | 40 | Bacillus |  | 0 |
|  | Bacillus infantis strain MER_TA_169 | 100 | 10 |  |  |  |
|  | Bacillus megaterium strain CD12 | 100 | 10 |  |  |  |
|  | Bacillus sp. (in: Bacteria) strain TH1 | 100 | 10 |  |  |  |
|  | Bacillus sp. (in: Bacteria) strain Y172001 | 99.93 | 10 |  |  |  |
|  | Bacillus sp. M71_D96 partial 16S rRNA gene, isolate M71_D96 | 100 | 10 |  |  |  |
|  | Bacillus thuringiensis strain NBIN-863 16S ribosomal RNA gene, partial sequence | 99.93 | 10 |  |  |  |
|  | Bacterium strain QLS201710OPB2 | 100 | 10 |  |  |  |
|  | Sporosarcina luteola strain 119A (C1TJ) | 99.93 | 10 | Unable to be detected |  | 0 |
|  | Unidentified ^b^ |  | 10 |  |  |  |
| 3 | Aerococcus sp. strain 201707CJKOP-Y34 | 100 | 10 | Aerococcus |  | 0 |
|  | Bacillus asahii strain OM18 chromosome, complete genome | 99.65 | 10 | Bacillus |  | 0 |
|  | Jeotgalicoccus sp. L39-1 16S ribosomal RNA gene, partial sequence | 99.79 | 550 | Jeotgalicoccus |  | 1.71 |
|  | Paenibacillus lactis strain LG2 | 99.93 | 10 | Paenibacillus |  | 0 |
|  | Paenibacillus sp. HX-1 | 100 | 10 |  |  |  |
|  | Paenibacillus taichungensis strain F9 | 99.65 | 10 |  |  |  |
|  | Staphylococcus saprophyticus strain 3-16 | 99.93 | >10^4^ | Staphylococcus |  | 1.34 |
|  | Staphylococcus sp. strain SS24 | 100 | 440 |  |  |  |
| 4 | Unidentified |  | 10 |  |  |  |
| 5 | Bacillus sp. (in: Bacteria) strain WH09-3 | 100 | 10 | Bacillus |  | 0 |
|  | Bacillus sp. BZ85 | 100 | 10 |  |  |  |
| 6 | Glutamicibacter arilaitensis strain MLS-4-4 | 100 | 10 | Unable to be detected |  |  |
|  | Terribacillus sp. EnB-bsy4 16S ribosomal RNA gene, partial sequence | 99.93 | 10 | Unable to be detected |  |  |
| 7 | Aerococcus sp. strain 201707CJKOP-Y34 | 100 | 50 | Aerococcus |  | 0 |
|  | Aerococcus viridans strain ML607 | 99.86 | 10 |  |  |  |
|  | Clostridium beijerinckii strain C-01 | 100 | 150 | Clostridium |  | 0.36 |
|  | Fictibacillus phosphorivorans strain FJAT-46891 | 99.93 |  | Unable to be detected |  |  |
|  | Lactococcus lactis strain W3-15 16S ribosonal RNA gene, partial sequence | 99.93 | >10^5^ | Lactococcus |  | 0 |
|  | Staphylococcus saprophyticus subsp. saprophyticus strain IAE105 | 100 | >10^4^ | Staphylococcus |  | 0 |
| 8 | Clostridium beijerinckii strain YBS3 | 100 | 10 | Clostridium |  | 0.19 |
| 9 | Bacillus pumilus strain CE92 | 100 | 10 | Bacillus |  | 0 |
|  | Bacillus sp. (in: Bacteria) strain H2 | 100 | 10 |  |  |  |
|  | Uncultured bacterium clone rRNA319 | 100 | 10 |  |  |  |
| 10 | Bacillus sp. strain FJAT-25513 | 99.86 | 10 | Bacillus |  | 0 |
|  | Fictibacillus halophilus strain FJAT-46947 | 99.93 | 30 | Unable to be detected |  |  |
| 11 | Bacillus megaterium strain FORT | 99.93 | 10 | Bacillus |  | 0 |
|  | Uncultured Massilia sp. clone b5 | 100 | 10 |  |  |  |
| 12 | Bacillus anthracis strain BJHS4 | 99.93 | 10 | Bacillus |  | 0 |
|  | Bacillus safensis strain K1 | 100 | 10 |  |  |  |
|  | Bacillus sp. (in: Bacteria) strain CCYL-4 | 100 | 10 |  |  |  |
|  | [Bacillus sp. (in: Bacteria) strain H2](https://blast.ncbi.nlm.nih.gov/Blast.cgi) | 100 | 10 |  |  |  |
|  | Bacillus sp. BG20 | 100 | 10 |  |  |  |
|  | Bhargavaea cecembensis strain HME8279 | 100 | 10 | Unable to be detected |  |  |
|  | Pseudoclavibacter sp. HP10L | 99.85 | 20 | Pseudoclavibacter |  | 0 |
|  | Staphylococcus hominis strain G1 | 100 | 10 | Staphylococcus |  | 0.84 |
| 13 | Bacillus pumilus strain CE92 | 100 | 170 | Bacillus |  | 0 |
|  | Bacillus sp. (in: Bacteria) strain TH1 | 100 | >104 |  |  |  |
|  | Staphylococcus epidermidis strain CG17 | 100 | 10 | Staphylococcus |  | 98.87 |
|  | Staphylococcus epidermidis strain CG17 | 100 | >104 |  |  |  |
|  | Staphylococcus epidermidis strain FDAARGOS_529 chromosome | 100 | 10 |  |  |  |
|  | Staphylococcus epidermidis strain M6 | 100 | 10 |  |  |  |
|  | Staphylococcus sp. strain A16 | 100 | 40 |  |  |  |
| 14 | Bacillus aerius strain UB02 | 100 | 10 | Negative |  |  |
|  | Bacillus sp. (in: Bacteria) strain PC-3 | 100 | 10 |  |  |  |
|  | Bacillus sp. (in: Bacteria) strain PC-3 | 100 | 10 |  |  |  |
|  | Bacterium BEL B9 | 100 | 10 |  |  |  |
|  | Fictibacillus sp. strain 201705CJKOP-93 | 100 | 10 |  |  |  |
| 15 | Bacillus oceanisediminis strain DSS-CAP-3 | 99.93 | 10 | Negative |  |  |
|  | Fictibacillus halophilus strain FJAT-46947 | 100 | 10 |  |  |  |
|  | Fictibacillus phosphorivorans strain S5 | 100 | 10 |  |  |  |
|  | Sporosarcina sp. strain AP1 | 100 | 10 |  |  |  |
| 16 | Bacillus sp. (in: Bacteria) strain CCYL-4 | 100 | 10 | Bacillus |  | 0 |
|  | Bacillus sp. (in: Bacteria) strain H2 | 100 | 10 |  |  |  |
|  | Bacillus sp. (in: Bacteria) strain TH1 | 100 | 10 |  |  |  |
| 17 | Bacillus firmus partial 16S rRNA gene, strain Bacillus sp. JC553 | 100 | 10 | Negative |  |  |
|  | Bacillus sp. (in: Bacteria) strain 1416X2 | 100 | 10 |  |  |  |
|  | Bacillus sp. N2a | 99.79 | 10 |  |  |  |
|  | Microbacterium foliorum strain 122 genome | 98.56 | 10 |  |  |  |
| 18 | Herbaspirillum huttiense strain SCSIO_43762 | 99.78 | >105 | Herbaspirillum |  | 0 |
|  | Uncultured firmicute partial 16S rRNA gene, clone Gitt-GS-63 | 99.57 | 10 |  |  |  |
| 19 | Bacillus oceanisediminis strain SCSIO_43758 | 100 | 10 | Bacillus |  | 0.59 |
|  | Bacillus pumilus strain CE92 | 100 | >105 |  |  |  |
| 20 | Bacillus amyloliquefaciens strain FS1092 | 100 | 10 | Bacillus |  | 0.43 |
|  | Bacterium strain GIm17 | 100 | 10 |  |  |  |
| 21 | Acetobacteraceae bacterium SAP1007.2 | 99.78 | 10 | Acetobacteraceae-Unclassified |  | 0 |
|  | Acidovorax sp. strain N15140 | 99.93 | 10 | Acidovorax |  | 0 |
|  | Agrococcus sp. B205-N2 | 99.86 | 10 | Aerococcus |  | 0 |
|  | Agrococcus terreus strain NA29 | 99.93 | 10 | Agrococcus |  | 0 |
|  | Bacillus altitudinis strain HTI 11 | 100 | 10 | Bacillus |  | 0.03 |
|  | Bacillus infantis strain BLB7 | 100 | 10 |  |  |  |
|  | Bacillus megaterium strain Q2 | 100 | 10 |  |  |  |
|  | Bacterium BEL B9 | 99.93 | 10 |  |  |  |
|  | Bacterium strain GIm11 | 100 | 10 |  |  |  |
|  | Herbaspirillum huttiense strain SCSIO_43762 | 99.78 | 10 | Herbaspirillum |  | 0 |
|  | Herbaspirillum sp. AU13533 | 100 | 30 |  |  |  |
|  | Sphingomonas sanguinis strain CGGJ9 | 100 | 30 | Sphingomonas |  | 0.08 |
|  | Staphylococcus warneri strain 22.1 chromosome | 99.86 | 10 | Staphylococcus |  | 0.06 |
|  | Uncultured microorganism clone cha-89 16S ribosomal RNA gene, partial sequence | 99.55 | 10 |  |  |  |
| 22 | Bacillus patagoniensis strain FJAT-45530 | 99.79 | 10 |  |  |  |
|  | Fictibacillus halophilus strain FJAT-46947 | 99.93 | 10 | Unable to be detected |  |  |
|  | Microbacterium hydrothermale strain COG-2 | 100 | 10 | Microbacterium |  | 0.03 |
|  | Microbacterium sp. S2SP302 | 100 | 10 | Microbacterium |  | 0.03 |
|  | Saccharopolyspora sp. strain R130 | 1 | 10 | Saccharopolyspora |  | 0 |
|  | Staphylococcus sp. strain R306 | 100 | 10 | Staphylococcus |  | 4.24 |
| 23 | Bacillus megaterium strain CS49 | 100 | 10 | Bacillus |  | 0.01 |
|  | Bacillus oceanisediminis strain DSS-ERY-3 | 100 | 450 |  |  |  |
|  | Bacillus sp. strain M4 | 100 | 10 |  |  |  |
|  | Bacillus subterraneus strain ZDC-01 | 100 | 10 |  |  |  |
|  | Brevundimonas vesicularis strain JH3 | 100 | 10 | Brevundimonas |  | 0 |
|  | Herbaspirillum huttiense strain SCSIO_43762 | 99.93 | 10 | Herbaspirillum |  | 0 |
|  | Kocuria sp. FB6 16S | 100 | 10 | Kocuria |  | 0 |
|  | Kocuria sp. strain JSM 1684078 | 100 | 40 |  |  |  |
|  | [Myrmecridium schulzeri strain PWQ2293](https://blast.ncbi.nlm.nih.gov/Blast.cgi) | 100 | 10 | Myrmecridium |  | 0 |
|  | Nigrospora sp. strain AQ14 | 100 | >105 | Nigrospora |  | 0 |
| 24 | Bacillus sp. (in: Bacteria) strain H2 | 100 | 10 | Bacillus |  | 0.08 |
|  | Bacillus sp. BG20 | 100 | 10 |  |  |  |
|  | Bacillus thaonhiensis strain FJAT-47807 | 100 | 10 |  |  |  |
|  | Curtobacterium sp. strain OTM104-1 | 100 | 10 | Curtobacterium |  | 0 |
|  | Microbacterium sp. KHA21 | 100 | 10 | Microbacterium |  | 0.26 |
|  | Roseomonas gilardii strain U14-5 chromosome 1 | 95.06 | 10 | Roseomonas |  | 0 |
|  | Terribacillus sp. EnB-bsy4 | 100 | 10 | Unable to be detected |  |  |
| 25 | Bacillus sp. (in: Bacteria) strain TH1 | 100 | 10 | Bacillus |  | 0 |
|  | Herbaspirillum huttiense strain SCSIO_43762 | 99.93 | 10 | Herbaspirillum |  | 0 |
|  | Herbaspirillum sp. AU13533 | 99.93 | 10 | Herbaspirillum |  | 0 |
|  | [Staphylococcus pasteuri strain PDS_PXHF_71](https://blast.ncbi.nlm.nih.gov/Blast.cgi) | 100 | 10 | Staphylococcus |  | 2.47 |
| 26 | Bacillus butanolivorans strain PHB-7a chromosome | 97.92 | 10 | Bacillus |  | 0.09 |
|  | Bacillus sp. (in: Bacteria) strain H2 | 100 | 10 |  |  |  |
|  | Bacillus sp. (in: Bacteria) strain TH1 | 100 | 10 |  |  |  |
|  | Kocuria sp. FB6 | 100 | 10 | Kocuria |  | 0.03 |
| 27 | [Bacillus sp. (in: Bacteria) strain TH1](https://blast.ncbi.nlm.nih.gov/Blast.cgi) | 100 | 10 | Bacillus |  | 0 |
|  | Staphylococcus hominis strain S2-6 16S ribosomal RNA gene, partial sequence | 100 | 10 | Staphylococcus |  | 2.65 |
| 28 | Arthrobacter gandavensis strain LR2314 | 99.93 | 10 | Arthrobacter |  | 0.09 |
|  | Bacillus megaterium strain CD12 | 100 | 100 | Bacillus |  | 0.15 |
|  | Bacillus megaterium strain PgKB16 | 99.66 | 10 |  |  |  |
|  | Bacillus megaterium strain SR7 chromosome | 100 | 200 |  |  |  |
|  | Bacillus safensis strain K1 | 100 | >104 |  |  |  |
|  | Bacillus sp. (in: Bacteria) strain H2 | 100 | 10 |  |  |  |
|  | Bacterium strain QLS201804OPB1 | 100 | 10 |  |  |  |
|  | Herbaspirillum sp. AU13533 | 99.93 | 10 | Herbaspirillum |  | 0 |
|  | Memnoniella sp. strain CNUFC-MSW24-2-12 | 99.79 | 10 | Memnoniella |  | 0 |
|  | Terribacillus goriensis strain MP602 | 100 | 20 | Unable to be detected |  |  |
|  | Uncultured fungus clone ZBJ201108-9 | 100 | 10 |  |  |  |
| 29 | Bacillus sp. H2-80 16S ribosomal RNA gene, partial sequence | 100 | 10 | Bacillus |  | 0 |
|  | Uncultured bacterium clone DM2-116 | 99.93 | 10 |  |  |  |
| 30 | Bacillus idriensis strain WTB13 | 100 | 10 | Bacillus |  | 0 |
|  | Bacillus siamensis strain K14 | 100 | >105 |  |  |  |
|  | Bacillus sp. strain JLT41 | 100 | 10 |  |  |  |
|  | Bacillus sp. strain SPB16 | 100 | 10 |  |  |  |
|  | Paenibacillus xylanexedens strain 3-4T | 100 |  | Paenibacillus |  | 0.08 |
|  | Staphylococcus sp. strain A16 | 100 | 10 | Staphylococcus |  | 0.59 |
| 31 | Agrococcus sp. B205-N2 | 99.85 | 20 | Aerococcus |  | 0 |
|  | Bacterium strain QLS201804OPB1 | 100 | 20 |  |  |  |
|  | Terribacillus saccharophilus strain MER_108 | 100 | 20 | Unable to be detected |  |  |
| 32 | Bacillus sp. (in: Bacteria) strain 201705CJKOP-33 | 99.86 |  | Bacillus |  | 0.09 |
|  | Bacillus sp. BG20 | 100 | 20 |  |  |  |
|  | Bacillus subtilis strain RQ6 | 100 | 10 |  |  |  |
|  | Exiguobacterium sp. GGC-D10A1 | 99.27 | 10 | Unable to be detected |  |  |
|  | Herbaspirillum huttiense strain SCSIO_43762 | 99.93 | 20 | Herbaspirillum |  | 0 |
|  | Rhodococcus sp. B7740 | 99.85 | 20 | Rhodococcus |  | 0 |
|  | Rhodococcus sp. YIM C683 | 99.85 | 10 |  |  |  |
|  | Staphylococcus haemolyticus strain 83131B chromosome | 100 | 10 | Staphylococcus |  | 0.5 |
|  | Terribacillus sp. EnB-bsy4 16S ribosomal RNA gene, partial sequence | 100 | 10 | Unable to be detected |  |  |
|  | Uncultured Bacillus sp. clone ONGS163 | 98.59 | 10 |  |  |  |
| 33 | Bacillus sp. M71_D96 partial 16S rRNA gene, isolate M71_D96 | 99.79 | 10 | Bacillus |  | 0 |
|  | Paenibacillus urinalis strain 38 | 99.65 | 10 | Paenibacillus |  | 0.26 |
|  | Psychrobacillus sp. strain SJ4 | 98.05 | 10 | Unable to be detected |  |  |
|  | Uncultured Paenibacillus sp. clone MJJ-21 | 99.79 | 10 |  |  |  |
| 34 | Bacillus pumilus gene for 16S ribosomal RNA, partial sequence, strain: CB | 100 | 20 | Bacillus |  | 0.49 |
|  | Bacillus sp. (in: Bacteria) strain GLS5 | 100 | 10 |  |  |  |
|  | Pseudomonas stutzeri strain SGAir0442 | 100 | 20 | Pseudomonas |  | 0.99 |
| 35 | Bacillus niacini strain X8 | 99.64 |  | Bacillus |  | 0 |
|  | Bacillus thaonhiensis strain FJAT-47807 | 100 | 20 |  |  |  |
|  | Bacterium strain GIm38 16S ribosomal RNA gene, partial sequence | 99.93 | 10 |  |  |  |
|  | Bacterium strain IMAU11955 16S ribosomal RNA gene, partial sequence | 100 | 10 |  |  |  |
|  | Paenibacillus sp. strain Avl-2 16S ribosomal RNA gene, partial sequence | 99.58 | 10 | Paenibacillus |  | 0 |
|  | Psychrobacillus psychrodurans | 99.93 | 10 |  |  |  |
|  | Psychrobacter alimentarius strain PAMC 27889 | 100 | 10 | Psychrobacter |  | 0.27 |
|  | Sporosarcina sp. S11-2 | 100 | 10 | Unable to be detected |  |  |
| 36 | Bacillus pumilus strain CE92 | 100 | 20 | Bacillus |  | 0.21 |
|  | Fictibacillus barbaricus strain ZSR22 | 99.72 | 10 | Unable to be detected |  |  |
| 37 | Bacillus licheniformis strain BpZL-4a | 100 | 10 | Bacillus |  | 0.01 |
|  | [Bacillus marisflavi strain NES-STR-2](https://blast.ncbi.nlm.nih.gov/Blast.cgi) | 100 | 10 |  |  |  |
|  | Bacillus sp. (in: Bacteria) strain CCYL-4 | 100 | 10 |  |  |  |
|  | Bacillus sp. strain SPB16 | 100 | 10 |  |  |  |
|  | Paenibacillus provencensis strain MER_78 | 99.93 | 10 | Paenibacillus |  | 0 |
|  | Paenisporosarcina quisquiliarum strain ICMP 22242 | 99.93 | 10 | Paenisporosarcina |  | 0 |
|  | Pseudomonas azotoformans strain F77 | 99.85 | 100 | Pseudomonas |  | 0.32 |
| 38 | Bacillus aerius strain UB02 | 100 | 10 | Bacillus |  | 0 |
|  | Bacillus sp. (in: Bacteria) strain H2 | 100 | 20 |  |  |  |
|  | Bacillus sp. PX25_S1 | 100 | 10 |  |  |  |
|  | Bacterium CSR-6 | 99.79 | 10 |  |  |  |
| 39 | Aerococcus sp. strain 201707CJKOP-Y34 | 99.93 | 10 | Aerococcus |  | 0 |
|  | Bacillus sp. (in: Bacteria) strain 201705CJKOP-76 | 99.93 | 10 | Bacillus |  | 0 |
|  | Bacillus sp. (in: Bacteria) strain CR16 | 99.93 | 10 |  |  |  |
|  | Bacillus sp. (in: Bacteria) strain TH1 | 100 | 10 |  |  |  |
|  | Bacillus subtilis strain 197WMS3 | 100 | 10 |  |  |  |
|  | Bacillus wiedmannii strain K3 | 100 | 10 |  |  |  |
|  | Sporosarcina sp. strain Hc026 | 100 | 10 | Unable to be detected |  |  |
|  | Staphylococcus sp. strain A16 | 100 | 10 | Staphylococcus |  | 1.32 |
| 40 | Bacterium 1C10 | 99.93 | 10 |  |  |  |
|  | Bacterium L7 16S ribosomal RNA gene, partial sequence | 99.93 | 10 |  |  |  |
|  | Bacterium strain HK-7 | 100 | 10 |  |  |  |
|  | Herbaspirillum sp. AU13533 | 99.93 | 40 | Herbaspirillum |  | 0 |
|  | Staphylococcus sp. strain CE01 | 100 | 10 | Staphylococcus |  | 0.13 |
|  | Uncultured bacterium clone nck274e02c1 | 99.53 | 10 |  |  |  |
|  | Uncultured bacterium partial 16S rRNA gene, clone MD03C07 | 99.93 | 10 |  |  |  |
| 41 | Elizabethkingia miricola strain EM798-26 chromosome | 99.80 | 10 | Elizabethkingia |  | 0 |
| 42 | Janthinobacterium agaricidamnosum NBRC 102515 = DSM 9628 | 96.47 | 20 | Janthinobacterium |  | 0 |
|  | Staphylococcus pasteuri strain BMLN9 | 100 | >105 | Staphylococcus |  | 2.75 |
| 43 | Bacterium strain GIm6 | 100 | 10 |  |  |  |
| 44 | [Bacillus infantis strain WTB98](https://blast.ncbi.nlm.nih.gov/Blast.cgi) | 99.86 | 10 | Bacillus |  | 0 |
|  | Herbaspirillum huttiense strain SCSIO_43762 | 99.93 | 10 | Herbaspirillum |  | 0 |
|  | Herbaspirillum sp. AU13533 | 99.93 | 10 |  |  |  |
| 45 | Bacillus sp. (in: Bacteria) strain PC-3 | 100 | 40 | Bacillus |  | 0 |
|  | Moraxella sp. strain M2_3 | 99.85 | 10 | Moraxella |  | 0 |
|  | Uncultured Brevibacillus sp. clone Y54 | 100 | 100 |  |  |  |
| 46 | Bacillus aerius strain UB02 | 100 | 10 | Bacillus |  | 0 |
|  | Bacillus altitudinis strain HTI 11 | 99.93 | 10 |  |  |  |
|  | Bacillus licheniformis strain NZ2-3 | 100 | 10 |  |  |  |
|  | Bacterium L7 16S ribosomal RNA gene, partial sequence | 99.93 | 20 |  |  |  |
|  | Bacterium strain GIm6 | 100 | 30 |  |  |  |
|  | Bacterium strain HK-7 | 100 | 30 |  |  |  |
|  | Staphylococcus aureus strain AT-A-4 | 99.86 | >104 | Staphylococcus |  | 2.57 |
|  | Staphylococcus epidermidis strain CG17 | 100 | >104 |  |  |  |
|  | Staphylococcus epidermidis strain FDAARGOS_529 chromosome | 99.93 | 200 |  |  |  |
|  | Staphylococcus sp. strain A16 | 100 | 400 |  |  |  |
| 47 | Herbaspirillum huttiense strain SCSIO_43762 | 99.93 | 10 | Herbaspirillum |  | 0 |
|  | Select seq MK414943.1 | 100 | 10 |  |  |  |
|  | Select seq MK696417.1 | 100 | 10 |  |  |  |
| 48 | Bacillus sp. 1P10SA | 99.72 | 100 |  |  |  |
|  | Select seq LS974830.1 | 100 | 10 |  |  |  |
|  | Staphylococcus epidermidis strain CG17 | 99.93 | >104 | Staphylococcus |  | 1.02 |
| 49 | Bacillus sp. (in: Bacteria) strain GR5 | 99.93 | 10 | Bacillus |  | 0 |
|  | Bacillus sp. BL04 16S | 99.86 | 10 |  |  |  |
|  | Bacterium 1C10 | 100 | 10 |  |  |  |
|  | Herbaspirillum sp. AU13533 | 99.93 | 10 | Herbaspirillum |  | 0 |
|  | Herbaspirillum sp. AU13533 | 99.93 | 10 |  |  |  |
|  | Microbacterium foliorum strain 122 genome | 97.52 | 10 | Microbacterium |  | 0.74 |
|  | Paenibacillus glucanolyticus strain B05 | 100 | 10 | Paenibacillus |  | 0 |
| 50 | Brevibacterium frigoritolerans strain WTB82 | 100 | 10 | Brevibacterium |  | 0 |
|  | Bacillus simplex NBRC 15720 = DSM 1321 | 99.86 | 10 | Bacillus |  | 0 |
|  | Staphylococcus capitis strain -Y41 | 100 | 1100 | Staphylococcus |  | 1.55 |

Abbreviation: colony forming units, CFU

^a^ The similarity of bacterial sequence cutoff is 97%.

^b^ If the genome sequence of a bacterium was identified as heterozygous three times or was difficult to bind, the bacterium is defined as unidentified bacteria.
