# Supplementary figures and images for: The pelvis urinary microbiome in patients with kidney stones and clinical associations
Source: BMC Microbiol. 2020 Nov 5;20:336. doi: 10.1186/s12866-020-01992-4 (PMC7643416; doi:10.1186/s12866-020-01992-4)

# Bladder A

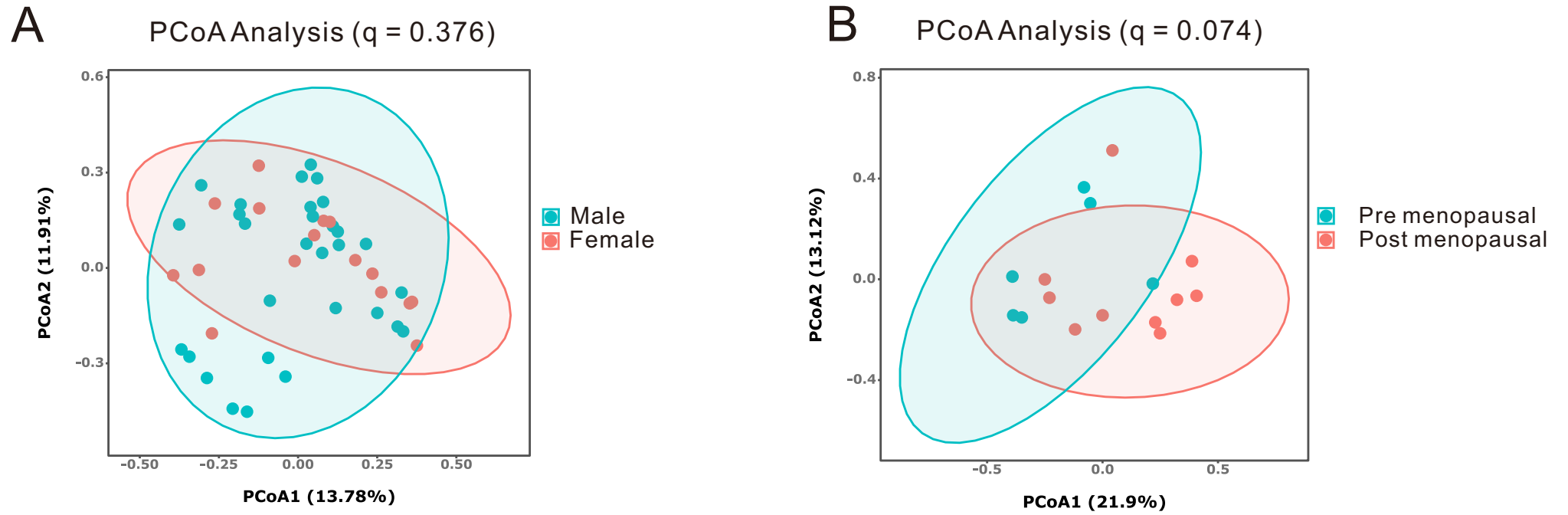

# SKP

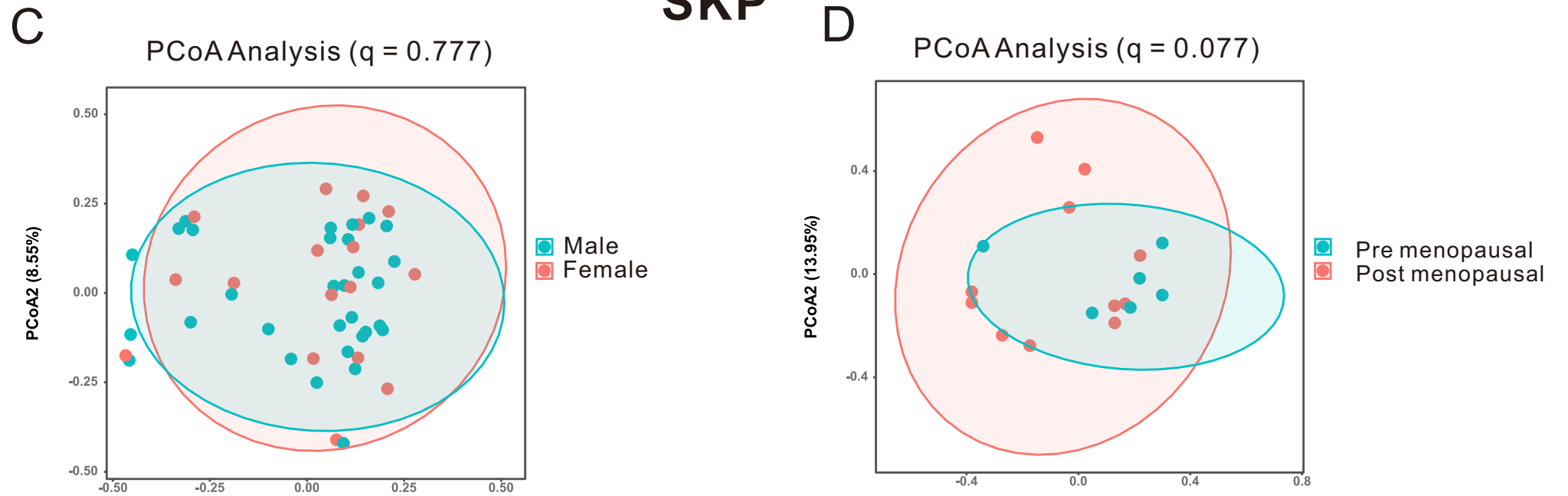

Supplement: Supplementary file 3 — Additional file 3: Figure S1. Bacterial structure between groups of males and females and pre menstrual and post menstrual females. PCoA shows the bacterial composition clustering of the groups based on Bray–Curtis distances, with each point corresponding to a patient and coloured according to the sample type of male or female in the SKP samples (A), pre menstrual and post menstrual females in the SKP samples (B), male or female in Bladder A samples (C), and pre menstrual and post menstrual females in the Bladder A samples (D). PERMANOVA indicated that the bacterial communities between males and females were not significantly different in the SKP samples (p > 0.05). [file 12866_2020_1992_MOESM3_ESM.pdf]

# Bladder A

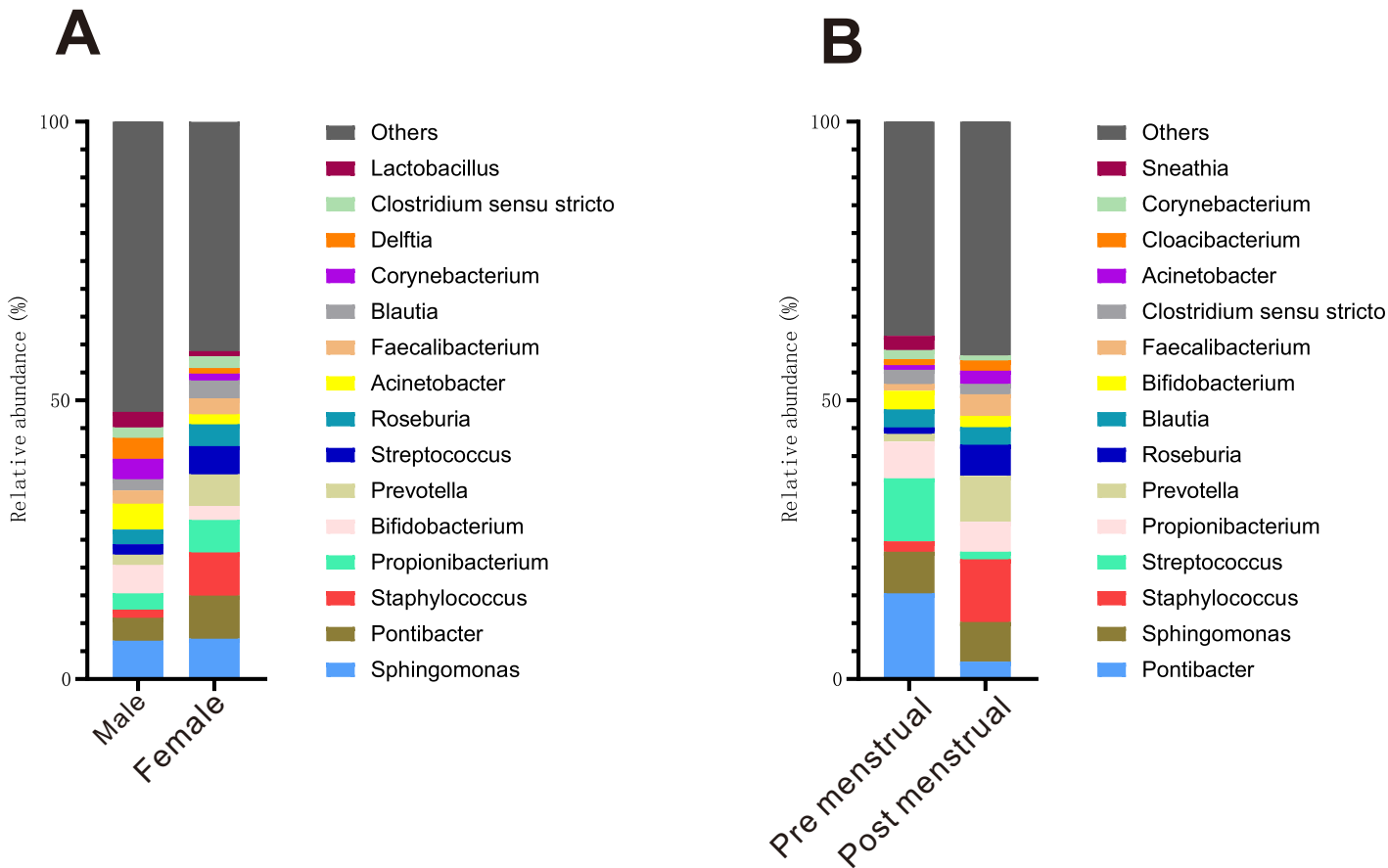

# SKP

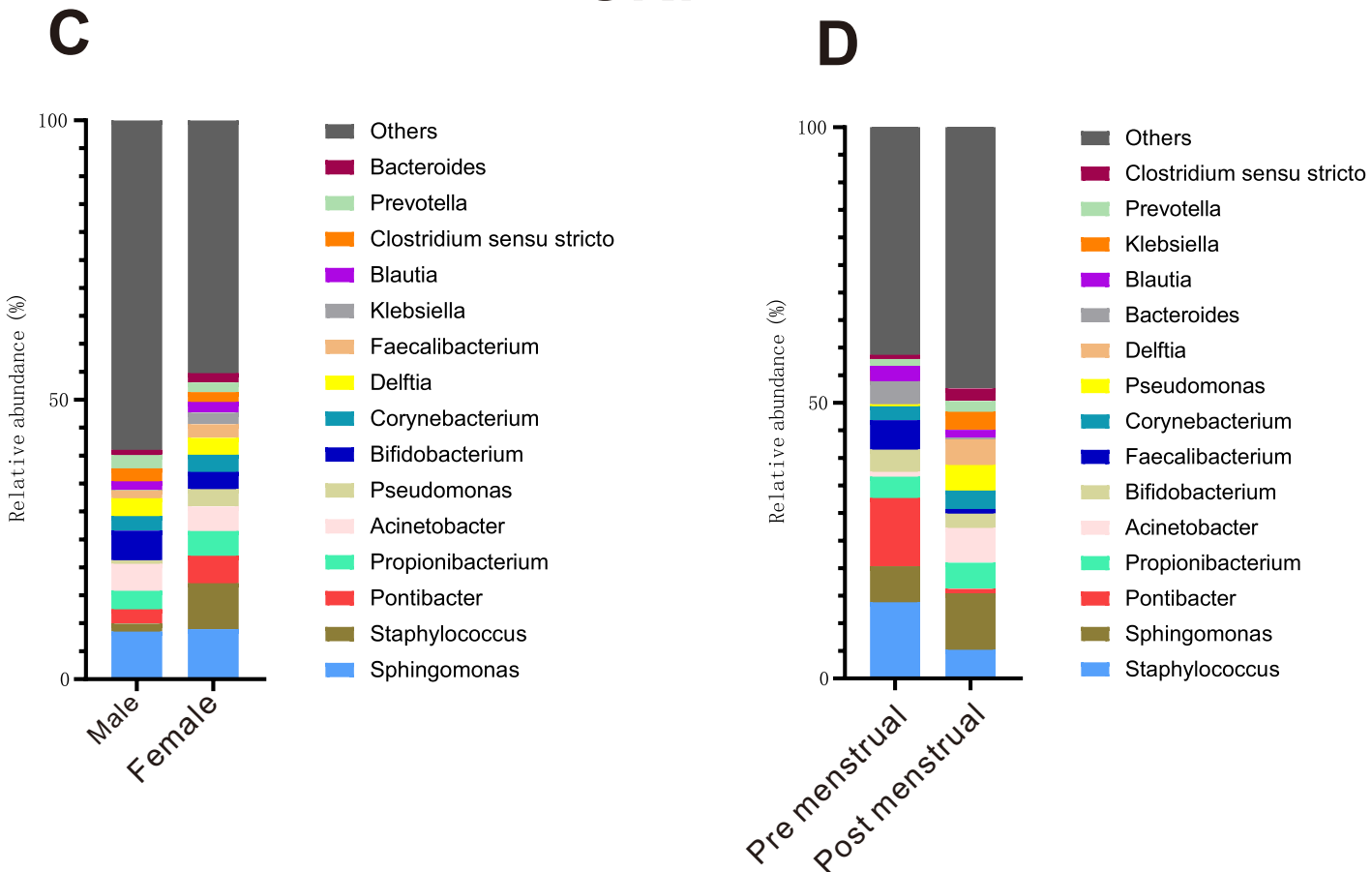

Supplement: Supplementary file 4 — Additional file 4: Figure S2. Bacterial genera distribution between the groups. The relative abundances of the major genera in males and females in the SKP samples (A), in pre menstrual and post menstrual females in the SKP samples (B), in males and females in the Bladder A samples (C) and in pre menstrual and post menstrual females in the Bladder A samples (D), as determined by 16S rDNA gene sequencing, in the SKP and NSKP samples were more similar to the Bladder A samples than the blood samples. “Others” includes all detected bacteria. [file 12866_2020_1992_MOESM4_ESM.pdf]
